# Diagnostics and Prevention of Occupational Allergy in Hairdressers

**DOI:** 10.1007/s11882-023-01076-z

**Published:** 2023-04-12

**Authors:** Wolfgang Uter, Jeanne D. Johansen, Jelena Macan, Cara Symanzik, Swen M. John

**Affiliations:** 1 Department of Medical Informatics, Biometry and Epidemiology, Friedrich-Alexander University of Erlangen-Nürnberg, Waldstr. 4-6, Erlangen, D-91054 Germany; 2 Department of Skin and Allergy, National Allergy Research Centre, University of Copenhagen, Gentofte Hospital, Copenhagen, Denmark; 3 Institute for Medical Research and Occupational Health, Zagreb, Croatia; 4 Institute for Interdisciplinary Dermatologic Prevention and Rehabilitation (iDerm), Osnabrück University, Osnabrück, Germany; 5 Department of Dermatology, Environmental Medicine and Health Theory, Osnabrück University, Osnabrück, Germany

**Keywords:** Hairdressers, Occupational diseases, Skin diseases, Asthma, Workers’ health, Hair cosmetics

## Abstract

**Purpose of Review:**

This study aims to provide an overview on current knowledge on occupational allergic diseases in hairdressers and up-to-date perspectives of prevention.

**Recent Findings:**

Hand eczema (dermatitis) is common in hairdressers, often caused by contact allergy to one or multiple small molecules (haptens) used, e.g., for dyeing, bleaching, and waving/relaxing or by ancillary substances such as preservatives. Hairdressers, compared to other patch-tested patients, have an up to fivefold increased risk to be found sensitized, e.g., against *p*-phenylenediamine, ammonium persulfate, and glyceryl thioglycolate. Some of these small molecules may induce respiratory sensitization causing allergic rhinitis and/or asthma, notably persulfate salts.

**Summary:**

Occupational hazards in hairdressing are well described. This knowledge needs to be put into use for risk reduction, mainly by substitution of allergenic ingredients by less allergenic ones, education, and use of ventilation and suitable single-use gloves.

## Introduction

Hairdressers regularly perform a variety of cosmetic procedures on their clients, such as washing, cutting and styling hair, waving or relaxing hair, and dyeing or bleaching hair, to name just the most common daily occupational tasks. Many salons offer additional services, e.g., make-up services, manicures and nail modeling, or hair and eyelash extensions. Thereby, hairdressers are heavily exposed to the corresponding (hair) cosmetic products [1•], in terms of skin contact and/or inhalational exposure. As the products used contain a number of small molecules which are well-known potent haptens, allergic contact dermatitis (ACD) is common in hairdressers, partly mixed with, or preceded by, irritant contact dermatitis (ICD), i.e., skin inflammation not involving the adaptive immune system. Consequently, hairdressers rank top in statistics on the incidence of occupational contact dermatitis, almost exclusively presenting as hand eczema (HE), followed by nurses and metal workers [2].

Focusing on the single chemicals responsible for ACD in hairdressers, patch test studies provide important evidence concerning those haptens regularly tested, assembled in a dedicated test series [3]. Additional, primarily anecdotal, evidence on other haptens causing ACD, or also special clinical presentations, is contributed by case reports. Contact allergy to important ingredients of hair cosmetics has recently been the subject of systematic reviews and meta-analyses which will be summarized below [4–6].

Unprotected or insufficiently protected skin is a prerequisite for delayed type hypersensitivity causing ACD but also for the rarer immediate-type hypersensitivity skin reactions, i.e., contact urticaria and protein contact dermatitis [7], possibly including angioedema and other systemic reactions. Finally, small molecules as well as large, usually proteinaceous molecules may not only cause immediate skin reactions, with or without systemic symptoms, but also respiratory disease, that is, rhinitis/conjunctivitis and asthma, e.g., to persulfate salts [8•].

The present review aims at providing an update on recent knowledge on the morbidity of allergic diseases and its causes in hairdressers, including recommendations for efficient diagnostic approaches, and followed by a presentation of a state-of-the-art toolbox for primary and secondary prevention. To this end, scientific literature published in 2018 or later has been considered (notwithstanding reference to selected core papers published before), including the series of systematic reviews on the topic mentioned above, rendering this review in parts a meta-review.

## Allergic Contact Dermatitis

Hairdressers are exposed both to mild, but cumulatively important skin irritants like detergents, and a multitude of potent haptens [9]. As they are all too often not sufficiently protected against these agents (e.g., by adequate glove use), such exposure leads to very frequent hand eczema (HE). As summarized in a recent systematic review, a pooled lifetime prevalence of 38.2% (95% confidence interval (CI) 32.6–43.8%) and a pooled 1-year prevalence of 20.3% (95% CI 18.0–22.6%) of HE was observed in hairdressers [10]. The pooled incidence rate of HE was 51.8 cases/1000 person-years (95% CI 42.6–61.0), and onset already during apprenticeship was very common [10]. These figures do not offer a distinction between ICD and ACD, possibly combined, as the underlying epidemiological studies usually did not include diagnostic patch testing. Moreover, atopic skin which is prone to barrier damage and inflammation is a risk factor, although the above meta-analysis concluded that the main risk factor of contact dermatitis in hairdressers is indeed occupational exposure [10]. This is in line with previous research identifying a much higher attributable risk of early-onset hand dermatitis associated with wet work exposure than with previous (atopic) hand eczema in hairdressing apprentices [11]. In conclusion, a very high risk of HE in this occupation is evident.

### Patch Testing

A specific diagnosis of ACD is generally only possible after identifying the causative hapten(s) by means of patch testing; a European guideline has been published [12•]. Many national contact dermatitis societies and suppliers of commercial patch test allergens maintain “hairdresser” or “hair cosmetic” series, and a European recommendation for such a patch test series exists [3]. In the diagnostic work-up of hairdressers with (potentially allergic) occupational contact dermatitis, this particular patch test series is combined with the so-called baseline series tested routinely in every patient being patch tested as well as products brought in by the patient, appropriately prepared for patch testing [13], if indicated [14]. International baseline series include *p*-phenylenediamine (PPD), used as primary intermediate in oxidative hair dyes, as well as several preservatives, which are included in basically all water-based hair cosmetics. A further commonly used primary intermediate is toluene-2,5-diamine (syn. *p*-toluenediamine (PTD)). The main hapten of bleaches is ammonium persulfate (APS), and for permanent waving or relaxing products, salts and esters of thioglycolic acid are commonly used, e.g., glyceryl thioglycolate (GMTG) or ammonium thioglycolate (ATG). Several other secondary intermediates of oxidative dyes and other substances potentially important as haptens are being patch tested; current results are presented in the following subsections.

### Oxidative Hair Dye Ingredients

In contrast to other procedures listed below, oxidative (permanent) hair dyeing is performed not only by hairdressers, but also by consumers (hairdressers’ clients or exclusive self-users), often starting at an early age for fashion reasons, and commonly performed later in life to conceal graying hair [15]. Hence, besides hairdressers, also a substantial number of consumers develop ACD to hair dye product ingredients. The prevalence of positive (allergic) patch test reactions to PPD has been quantitatively summarized in a recent meta-analysis [6]. The overall pooled prevalence in consecutively patch-tested patients has been estimated to be 4.31% (95% confidence interval (CI) 3.79–4.91%), whereas on the population level, it has been estimated to be 0.89% (95% CI, 0.56–1.41%). The relative risk of being tested positive to PPD being a hairdresser compared to not being a hairdresser has been estimated to be 5.38 (95% CI, 3.95–7.33). An analysis of risk factors related to PPD exposure based on 271 allergic reactions diagnosed in 4314 patients 2008–2013 found that among those positive, 80% had their hair dyed, about half of these subsequently developing scalp dermatitis, whereas only 11% had had a henna tattoo (these tattoos are often adulterated with PPD in high concentrations to achieve a longer-lasting color and thus highly sensitizing). A more detailed analysis revealed hair dyeing as the main risk factor with an odds ratio (OR) of 6.0 (95% CI, 3.9–9.4), followed by henna tattoos (OR 2.4; 95%CI, 1.5–3.7) and being a hairdresser (OR 2.1; 95%CI, 1.3–3.2), respectively. Interestingly, neither dyeing of own hair nor application of a temporary henna tattoo seemed to affect PPD sensitization in hairdressers [16].

In contrast to PPD, PTD is generally patch tested in a “hair cosmetic series,” where a high share of positive reactions both in hairdressers and consumers is usually seen. A selection of current test results according to the recommended hair cosmetic series [3] is shown in Table 1. *p*-Aminophenol as another primary intermediate and several secondary intermediates (“couplers”) are well-known allergens (Table 1). Derivatives of PPD with side chains introduced to lessen allergenic potency while maintaining technical usability have been introduced to the market, such as 2-methoxymethyl-PPD (me-PPD). According to quantitative risk assessment, the margin of safety for induction of sensitization should have improved by a factor of at least 20 by this modification, compared to PPD or PTD [17]. However, clinical epidemiological results to confirm or dispel the notion of an improved safety with regard to sensitization are yet awaited for.

**Table 1:** Patch test recommendation for a core “hair cosmetic series” (taken from [3]), *p*-phenylenediamine (PPD) being included in “baseline series,” and important ancillary substances with some selected current results (Italy 2020 [27], Greece 2020 [54], North America 2022 [19], and central Europe 2023 [55]). aq, water; pet, petrolatum

Allergen	Italy (2020)	Greece (2020)	North America (2022)	Central Europe (2023)
Nr. tested	157	362^c^	3481^d^	920^b^
**Oxidative hair dye ingredients**				
*p*-Phenylenediamine 1% pet	18.5%	52.2%	35.8%	19.7%
Toluene-2,5-diamine 1% pet	7.9%	38.7%		20.0%
p-Aminophenol 1% pet	4.1%	26.0%		5.0%
m-Aminophenol 1% pet	2.3%	19.3%		3.4%
Resorcinol 1 or 2% pet	0.7%	0.3%		0.0%
p-Methylaminophenol (sulfate) 1% pet				6.8%
**Bleach ingredient**				
Ammonium persulfate 2.5% pet	13.6%	18.2%	0.5%	14.4%
**Waving/relaxing agents**				
Ammonium thioglycolate 1 to 2.5% aq.^a^	1.4%			1.8%
Glyceryl thioglycolate 1% pet	2.1%	0.55%	4.4%	3.9%
**Ancillary substances**				
MI 0.2% aq. or MCI/MI 0.02% aq		3.0%	9.7%	10.5%
Cocamidopropyl betaine 1% aq		8.6%	8.7%	2.2%

^a^Patch test material freshly prepared from stock solution is preferable

^b^Hairdressers with suspected (occupational) ACD as denominator

^c^Patients patch tested for suspected ACD to hair cosmetics as denominator

^d^Patients with positive patch test reactions related to hair cosmetics as denominator

### Bleach Ingredients

Although contact sensitization to APS is rare in consecutively patch-tested patients, e.g., 1.8% in 10526 North American patients tested 2015–2019 [18], and even in those presenting with hair cosmetic-related ACD [19], hairdressers have been found to be sensitized much more commonly. Based on results from three central European and one Japanese study, a recent meta-analysis has estimated a 5.25-fold (95% CI, 2.09, 13.14) increased risk in hairdressers to be sensitized to APS compared to other patients, even if patch tested for suspected hair cosmetic-related ACD [6]. Among 55 hairdressers with occupational ACD patch tested in Istanbul, Turkey, 27 (49.1%) were allergic to APS [20]. The vastly differing prevalences for APS evident from Table 1 are most likely a consequence of different patient selection.

### Waving/Relaxing Agents

A permanent change of the hair structure is achieved by application of agents acting on the disulfide bonds of hair keratin. These include the “classical” ammonium thioglycolate (ATG), which is a rare allergen. By contrast, the glyceryl ester of thioglycolic acid (glyceryl thioglycolate (GMTG)) is a relatively strong allergen, although, after a historical “epidemic” among German hairdressers and a subsequent withdrawal and later ban of this compound in Germany [21], current sensitization prevalence among hairdressers is quite low (Table 1). Besides these “classical” waving agents, cysteamine hydrochloride (HCl) is used for perming. In a Japanese case series, 7 of 17 hairdressers consulting the Yamaguchi clinic with ACD, cysteamine HCl 0.5 and 1% in pet., respectively, were positive, as were open tests with the developer solution in 5 of 6 patients thus tested [22]. Another alternative waving agent is thiolactic acid and its ammonium salt; current evidence on the frequency of contact sensitization is lacking, after a previous study found these agents rare allergens, if at all, and very difficult to patch test owing to their irritation potential [23].

### Cosmetic Glues

Some hair treatments involve glues, e.g., for fixing hair extensions. Such cosmetic glues might also be used for eyelash extension applications or when performing nail modeling in hairdressing salons. Although cyanoacrylate glues have been reported to cause contact allergy and are indeed found in these specific products, only a few cases in clients, but hitherto none in hairdressers have been published [4]. By contrast, (meth-)acrylate glues and resins used for nail modeling are a well-known cause of contact allergy in those hairdressers involved in such cosmetic activity, which is otherwise also offered as sole professional activity by nail designers. No matter which affiliation, the risk for contact allergy, e.g., to 2-hydroxyethyl methacrylate (2-HEMA), is estimated to be higher by a factor of 8.47 (95% CI, 4.70–15.3) compared to other professions or exposures, according to a recent meta-analysis [4]. Interestingly, acknowledging sensitization risk by possibly less diligent self-application, 2-HEMA has recently been restricted to be used by professionals only in the EU, based on an opinion of the Scientific Committee on Consumer Safety (SCCS/1592/17 and regulation EU 2020/1682). However, while possibly offering some benefit for consumers, this restriction evidently does not target professional users, that is, a significant share of hairdressers and the specialized nail designers. Moreover, other (meth-)acrylates are in use which exhibit a similar sensitization risk [24]; thus, “no touch” techniques and adequate glove wearing which helped to reduce sensitization risk elsewhere, e.g., in dental technicians, are certainly warranted for nail modeling, too [25].

### Auxillary Ingredients

This diverse group of hair cosmetic product ingredients includes mostly preservatives, emulsifiers, and fragrances, all not specific for this range of products, but abundant in these. Among the emulsifiers, cocamidopropyl betaine (CAPB) has been patch tested most often, enabling a reliable comparison between hairdressers and non-hairdressers. The relative risk estimated in a recent meta-analysis was 1.71 (95% CI, 1.29–2.27) [5], indicating a slightly but significantly increased risk; for some further results, see Table 1. In view of the fact that hairdressers’ skin is much more often exposed, compared to consumer exposure on which risk assessment is based [1•], preservatives and other such “everyday ingredients” seem to lead to an excess risk of contact allergy in hairdressers — if no protective gloves are worn, which is very often the case. Accordingly, a significantly increased risk associated with hairdressing has been found for methylchloroisothiazolinone (MCI)/methylisothiazolinone (MI) (2.26; 95% CI, 1.67–3.07) and methyldibromo glutaronitrile (MDBGN; 1.88; 95% CI, 1.28–2.76), according to a recent meta-analysis [26]. As an example, 6 (10.9%) of 55 hairdressers with occupational ACD patch tested in Istanbul, Turkey, were found sensitized to MCI/MI or MI [20].

### Tools, Personal Protective Equipment, and Skin Care

Historically, nickel has been considered a “hairdresser allergen,” as nickel allergy was very common among hairdressers. These being predominantly female and relatively young, however, nickel allergy prevalence simply reflected background nickel allergy prevalence in this gender/age stratum; hence, a suitably adjusted or controlled analysis will not reveal a significant difference [27]. Nevertheless, nickel (or cobalt) may be released in relevant amounts from tools such as scissors, clips, and tweezers [28, 29] — although at least nickel release by work tools has been restricted by national regulations [21] and also generally in Europe (Directive 94/27/EC, now covered by item 27 of Annex XVII to REACH) — which may lead to ACD in case of pre-existing sensitization or even to de novo sensitization.

Owing to the need to wear protective gloves (see “Prevention” below), hairdressing entails a certain risk of contact allergy to rubber accelerators (vulcanizing agents) as residues from the glove production process. Indeed, a slightly but significantly increased risk of contact allergy to accelerators has been found in a recent meta-analysis concerning thiuram mix (RR 1.61, 95% CI, 1.12–2.32) and 2-mercaptobenzothiazole (RR 2.68, 95% CI, 1.15–6.23) [26]. This finding certainly corroborates the need to provide gloves with an as low as possible allergen content. The same holds true for ingredients of after- or at-work emollients, such as preservatives, emulsifiers, and fragrances. In a registry-based analysis of occupational dermatitis cases in the UK, a strikingly higher-than-average share of fragrance allergy had been noted among hairdressers [30] pointing to an increased risk of sensitization to these ubiquitous allergens.

## Immediate-Type Skin Reactions

In a Danish registry-based study covering 2006 to 2011, among 381 hairdressers with recognized occupational contact dermatitis, 19 were diagnosed with occupational contact urticaria (OCU); 16 apprentices and 3 fully trained. In seven of these, this was caused by APS, in one by potassium persulfate (PPS), in 4 by APS and PPS, and one by a non-specified bleach [31]. Among 290 hairdressers with occupational dermatitis diagnosed 2005–2018 at the Finnish Institute of Occupational Health, Helsinki, 15 had OCU, thereof 11 to persulfate salts, one to henna (*Lawsonia inermis*), and three to hair products not further specified [32]. The incidence of OCU in Finnish hairdressers has been estimated to be 0.91 (95% CI, 0.53–1.47)/100,000 person-years [33•].

## Respiratory Sensitization

Concerning the inhalational occupational exposure in hairdressers, it is evident that exposure to various respiratory irritants is common, particularly to ammonia and volatile organic compounds, which often exceed occupational threshold limit values [34•, 35]. Respiratory sensitization is commonly considered for persulfate salts contained in bleaching products. Persulfate salts (PS), namely, ammonium and potassium persulfate (APS and PPS, respectively), are very well-established low-molecular-weight (LMW) occupational asthmogens and the main cause of occupational rhinitis and asthma in hairdressers [8•]. However, pathophysiological mechanism(s) of respiratory inflammation caused by PS are not yet clarified. Beside non-specific immunological pathways including chemical irritation with possible epithelial damage and histamine liberation, specific immunological reactions like IgE- or non-IgE-mediated immediate types of allergic reactions were suggested [8•, 36]. Diagnostic procedures in this respect are, so far, hampered by the lack of commercially available standardized APS/PPS preparations for skin prick testing (SPT), and standardized reagents and methods for measuring specific IgE to PSs. An SPT is performed with fresh “in-house” preparations of APS/PPS, and freshness was pointed out as relevant for SPT result [37]. Hougaard et al. in 2012 proposed a guidance for preparation of SPT material from APS/PPS [38]. SPT results vary between studies, mostly being negative in hairdressers with respiratory disorders. Recently, among 148 hairdressers seen in a German occupational medicine university clinic between 2012 and 2019, positive SPT to APS with clinical relevance was found in 15% [36]. Specific IgE antibodies to PSs have been measured even more rarely, only with “in-house” methods, and yielding positive results sporadically [37, 39]. Therefore, specific inhalational nasal or bronchial challenge with PS remains the diagnostic gold standard for the confirmation of causal role of PS exposure for respiratory disease. Most studies pointed out the development of late bronchial responses with eosinophilic inflammation during the positive specific bronchial challenge with PS [8•]. Some studies describe the co-existence of positive patch test and SPT to APS/PPS with concomitant skin and respiratory symptoms [8•]. In these cases, skin symptoms usually occur before the respiratory symptoms. Further clinical research of this aspect is of great interest, as animal experiments support the notion that primary skin exposure to LMW asthmogens may provoke systemic sensitization and asthma development after secondary inhalational exposure [40•]. Among patients with occupational asthma, exposure to LMW asthmogens was associated with a high frequency of contact dermatitis [41].

Other hairdressing chemicals are rarely described as a potential cause of respiratory allergy in hairdressers. A well-documented case of rhinitis and asthma was accompanied by contact urticaria owing to immediate-type sensitization to two different types of “henna products,” namely, *Cassia obovata* (yellow henna) and *L. inermis* (red henna), as diagnosed by positive skin prick tests and nasal provocation, but negative bronchial provocation testing [42]. Two Finnish beauticians had applied eyelash extensions for several hours every day and developed occupation-related rhinitis and one also bronchial asthma. Challenge tests revealed low levels of ethyl cyanoacrylate of 0.4 mg/m^3^ as a cause [43] of these presumably rare problems related to intense exposure.

## Prevention

For the prevention of allergic diseases in hairdressers, the so-called STOP principle is applied as in basically any other occupational health and safety context (see Table 2 and [26]). According to this well-founded hierarchical principle, the “S” in STOP stands for substitution which is regarded as the top-priority measure; the other measures would only be considered if substitution is not possible. Besides the ban of GMTG on a national level in Germany [21], development and marketing of PPD derivatives with a lesser sensitization potency, such as me-PPD [17], can be regarded as a substitution effort. The “T” in STOP is for technical measures. In a personal service work context, technical measures to limit hapten or allergen contact are somewhat limited compared to the producing industries where use of robots and encapsulated processes can often be implemented to avoid skin or respiratory hazards. However, sufficient ventilation and use of an exhaust ventilation are a necessity in case hazardous products are handled which liberate, e.g., APS or other persulfate salts in dusting bleaching powder still in use [44], or even formaldehyde used for hair forming [45]. If neither substitution nor technical measures are feasible, organizational measures, the “O” in STOP, shall be implemented. In the hairdressing trade, this mainly implies that unavoidable wet work must be distributed as fair as possible among several employees in order to reduce exposure for the individual. Only as an ultimate level of prevention, the “P” in STOP, personal measures, must be taken if all other measures are either not feasible or insufficiently effective. In hairdressing, personal protective equipment mainly includes provision and use of suitable protective gloves when exposure to hazardous product demands. As protective glove material, synthetic rubber offers the best protection (for example, nitrile gloves), although polyvinyl chloride (PVC) gloves may be sufficient in most cases [46]. The importance of as low as possible residual chemical levels [46] to minimize sensitization risk to these has already been stressed above. Further aspects are included in Table 2.

**Table 2:** The hierarchical “STOP” principle of prevention with some examples pertaining to hairdressers’ exposure to haptens and allergens, respectively [26]

	Principle	Examples
**S**	Substitution	GMTG was banned and has been replaced by other waving agents. This reduced GMTG sensitization in hairdressers
		Dusting hair bleaching powders were mostly substituted by dust-free bleaching powders or bleaching creams, reducing respiratory exposure
**T**	Technical measures	Suitable room ventilation, e.g., at least 100 m^3^/employee
		For mixing and decanting work, specially designated workplaces are needed if hazardous gases, vapors, or suspended matter occur during handling
**O**	Organizational measures	Even/fair distribution of “wet work” tasks among all employees
**P**	Personal measures	Suitable protective gloves^a^ must be made available to employees for the several activities^b^
		A skin protection plan must be displayed in a clearly visible place in every hairdressing salon

*GMTG* glyceryl thioglycolate

^a^(i) impervious to hairdressing chemicals (low chemicals hazards protection required), (ii) extending above the wrist, (iii) low-sensitizing, (iv) single-use gloves must not be re-used

^b^(i) head massage; (ii) coloring, tinting, and bleaching; (iii) perming/relaxing, including trial wrapping and fixation; (iv) preparation, mixing, and decanting of hazardous substances; (v) hair washing; and (vi) wet cleaning or disinfection of work equipment, devices, tools, and rooms

Primary prevention has different starting points in terms of stakeholders involved:

- Regarding substitution, industry and related research and regulators have a crucial role; in this context, it is worth noting that in Europe, risk assessment contributed by the Scientific Committee on Consumer Safety (SCCS) and its predecessors generally focuses on consumer and usually not on occupational safety. Hairdressers, however, apply hair (or nail) cosmetic products much more frequently than consumers [1•] and are thus not adequately covered by this institution [47]. The US Cosmetic Ingredients Review (https://www.cir-safety.org/) in its set of rules and procedures does not seem to formally and regularly address occupational aspects of cosmetic product exposure either.
- Hairdressers need to wear personal protective equipment (meaning mainly gloves); this is inevitable for effective primary (as well as secondary) prevention. Firmly integrating knowledge on, and practice of, safe working techniques into professional training and exams is important and has been proven to be highly effective in reducing the incidence of occupational hand dermatitis (ICD and/or ACD) in Denmark [48, 49••].
- In the salons, owners should continually perform a risk assessment, selecting and implementing feasible preventive measures along the STOP principle together with workers and health professionals. This process can be supported by risk assessment tools, e.g., offered by EU OSHA (https://oiraproject.eu/en/oira-tools/hairdressers, available in the EU languages).

Secondary prevention builds on all the above-mentioned activities and aims to prevent disease progression in an already affected individual hairdresser, relying on a number of further prerequisites:

- Easy and timely access to adequate medical care of affected hairdressers, who normally work in small enterprises without regular visits of, or access to, occupational physicians or hygienists.
- Medical care, normally by dermatologists in case of skin problems [50, 51], and ENT specialists, allergists, or lung specialists (https://www.worldallergy.org/education-and-programs/education/allergic-disease-resource-center/professionals/diagnosis-of-occupational-asthma) in case of respiratory diseases must include comprehensive testing, either patch testing for suspected ACD and/or skin, laboratory, or provocation tests to confirm immediate-type hypersensitivity.
- Structured seminars to refresh or create knowledge on causes and prevention of occupational dermatitis have proven helpful to achieve sustained remission of occupational disease and thus avoid loss of job, retraining, workers’ compensation, and other individual or societal costs.
- An in-patient course of (supplemental or repeat, as indicated) diagnostics and treatment, combined with above seminars, has helped to reduce the individual as well as economic impact in more recalcitrant cases not amenable to first-line outpatient care [52, 53].

## Conclusion

**Fig. 1:**
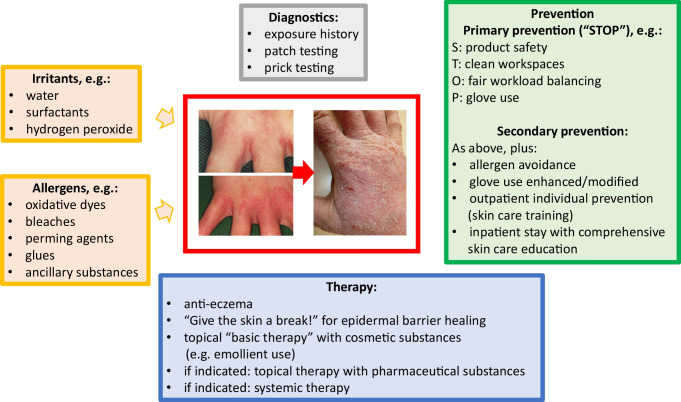
Factors associated with the etiology and prognosis of hand eczema in hairdressers

Contact allergens (haptens) and, to a lesser extent in terms of incidence, allergens causing immediate-type hypersensitivity reactions are an important cause of occupational allergic disease in hairdressers. New fashion trends such as artificial nail modeling evolve and create new risks, the consequences of which need to be adequately diagnosed and carefully monitored. Concerning prevention, several building blocks of primary and secondary prevention exist and have proven useful for reducing the burden of hazardous exposure and disease, respectively. Fully implementing such best-practice components in (inter-)national prevention schemes should further spread and exploit their benefit, followed up by suitable research targeting further amendments, where necessary (Fig. 1).

## Data Availability

This is a review, where no actual data were used, but just publications (all theoretically accessible according to a reader's (institution) licensing). Hence it seems unnecessary to include a data availability statement.
